# Does IRISIN Have a BRITE Future as a Therapeutic Agent in Humans?

**DOI:** 10.1007/s13679-014-0091-1

**Published:** 2014-02-02

**Authors:** Brian A. Irving, Christopher D. Still, George Argyropoulos

**Affiliations:** 1 Department of Gastroenterology, Geisinger Medical Center, Danville, PA USA; 2 Geisinger Obesity Institute, Geisinger Medical Center, Danville, PA USA; 3 Weis Center for Research, Geisinger Medical Center, Danville, PA USA; 4 Geisinger Obesity Institute, Geisinger Health System, 100 N. Academy Ave, Danville, PA 17868 USA

**Keywords:** Adaptive thermogenesis, Uncoupling, Physical activity, Weight loss, Myokines, Adipokines, FNDC5, Metabolism, Human, Mice, Brown adipose tissue, White adipose tissue, Beige adipose tissue, Skeletal muscle, Mitochondria, UCP1, Irisin, Brite adipocytes

## Abstract

The epidemic of obesity has contributed to the rapid rise in comorbid conditions such as cardiovascular disease, type 2 diabetes, sleep apnea, and hypertension among others. Therefore, there is a critical need to develop therapeutic strategies to reduce the prevalence of the disease. Skeletal muscle cells secrete signaling cytokines/peptides (referred to as myokines) that act in autocrine, paracrine, and endocrine fashion. Myokines have been hypothesized to contribute to the immediate and chronic benefits of exercise and may thus serve as attractive therapeutic agents for the treatment of obesity. The recent discovery of the irisin, a proposed myokine, has gained much attention over the last two years as a potential therapeutic agent. Preliminary studies demonstrated that irisin has the potential to induce “browning” of white adipocytes in mice. If these findings in mice could be translated to humans, irisin could be a potential therapeutic agent for the treatment of obesity. Limitations with the available antibodies, however, have raised concerns regarding the detectability of irisin in circulation. Moreover, the gene encoding irisin, *FNDC5*, is expressed robustly not only in muscle but also in various white adipose tissues (WAT) in humans, raising the possibility for increased thermogenesis through autocrine mechanisms. Here we will discuss the browning of WAT, the discovery of irisin, and its potential role in improving metabolic health in humans.

## Introduction

Obesity has rapidly become a worldwide epidemic. In parallel, the prevalence of obesity-related comorbid conditions has also escalated, including insulin resistance, metabolic syndrome, type 2 diabetes, hypertension, chronic kidney disease, cardiovascular disease, heart failure, cancer, and dementia [1–3]. As expected, a recent meta-analysis from the US Centers for Disease Control and Prevention confirms that obesity is associated with increased all-cause mortality [4]. The rapid increase in obesity and obesity-related comorbid conditions has coincided with the rapidly changing landscape of our obesogenic environment [5]. In particular, it has coincided with the systemic reductions in total daily physical activity as well as reductions in vigorous physical activity [5, 6]. Exercise has long been recognized for its pluripotent effects on body composition [7, 8], metabolic health [9], cardiovascular disease [10, 11] and mental health [12]. The underlying mechanism(s) for the clinical benefits of exercise remains to be fully elucidated. Over the past decade, it has become increasingly recognized that skeletal muscle cells secrete signaling cytokines/peptides that act in autocrine, paracrine, and endocrine fashion in response to skeletal muscle contraction (e.g., exercise) [13]. The secreted cytokines/peptides, referred to as myokines, have been hypothesized to contribute to the immediate and chronic benefits of exercise [13]. The recent discovery of irisin by Bostrom et al. [14••], a putative exercise induced myokine, that is credited for improving metabolic health by its ability to brown white adipose tissue (WAT) in mice has received considerable attention over the last two years. Although it remains to be determined whether irisin has the ability to brown WAT and improve metabolic health in humans, it represents a potentially attractive therapeutic agent for treating obesity and metabolic disease in humans. Here we will briefly discuss the browning of WAT, the discovery of irisin, and the potential role that irisin may play in browning WAT and improving metabolic health in humans.

## Browning of White Adipocytes

Classically, adipose tissue is characterized as either WAT or brown adipose tissue (BAT). Adipocytes from WAT serve as the primary site for lipid storage; whereas adipocytes from BAT are highly specialized cells designed to produce heat through uncoupled respiration that leads to concomitant dissipation of energy [15]. The physical, metabolic, and regulatory characteristics of WAT and BAT have been extensively reviewed elsewhere [16–21]. In brief, adipocytes from WAT have a unilocular lipid droplet, few mitochondria, and a relatively low metabolic rate [21]. In contrast, adipocytes from BAT have multilocular lipid droplets, many mitochondria, and a relatively high metabolic rate [21]. The relatively high metabolic rate observed in BAT compared to WAT is due to the presence of uncoupling protein 1 (UCP1), which is negligibly expressed in WAT [21].

The presence of brown adipocytes in WAT has been known for many years. Young et al. [22] were the first to report the presence of brown adipocytes in WAT of female BALB/c mice following cold acclimatization. Subsequently, brown adipocytes were identified in multiple WAT fat pads in rats [23]. Enrichment and activation of BAT represents an attractive therapeutic strategy to combat obesity and metabolic disease. The presence of UCP1 positive cells in WAT can also be pharmacologically enriched by β-adrenergic stimuli [23–25] as well as PPARγ agonist [26–28]. Recent evidence has demonstrated that the brown adipocytes (i.e., UCP1 positive cells) found in WAT are actually a distinct sub-population of white adipocytes (referred to as brown-in-white (brite) or beige adipocytes) [15, 26]. Co-culture experiments demonstrated that beige/brite adipocytes treated with rosiglitazone (a PPARγ agonist) can be induced to differentiate into adipocytes with thermogenic potential in the absence of classical brown adipocyte-specific markers (e.g., Zic2, Lhx8, Meox3, and PRDM16) [26]. In addition, these beige/brite adipocytes are also characterized as having the white adipocyte-specific marker Hoxc9 while lacking the white adipocyte-specific marker tcf21 [26]. Taken together, these results indicate that the beige/brite adipocytes are truly a distinct subtype of white adipocytes with potentially hidden capacity for higher metabolic rate [26]. In 2012, Wu et al. [15] confirmed that the beige/brite adipocytes found in WAT depots in mice and humans are in fact distinct from classical brown adipocytes that are more abundant in mice as a specialized depot [19, 29]. Specifically, for the first time they demonstrated that the beige/brite adipocytes emerge from non-myf-5 progenitor cells, in contrast to brown adipocytes, which are derived from myf-5 positive progenitor cells [15]. The exquisite regulation of the “browning” of white adipocytes in response to environmental, hormonal, and metabolic stimuli is quite remarkable. Excellent reviews of the development and regulatory control of beige/brite adipocytes have recently been published [19, 29]. Similar to BAT, enrichment and activation of beige/brite adipocytes represents an attractive therapeutic strategy to combat obesity and metabolic disease. The recent discovery of irisin and its potential to induce “browning” of white adipocytes has gained much attention over the last two years [14••], which will be discussed in detail below.

## Discovery of Irisin

It is well accepted that exercise is the corner stone of a healthy lifestyle and the frontline defense for primary and secondary prevention of many metabolic and cardiovascular diseases. Chronic endurance training has also been shown to induce skeletal muscle mitochondrial biogenesis [30, 31], which is regulated by the expression and activity of PPAR-γ co-activator-1α PGC1-α [31–33]. Indeed, the canonical PGC1-α (i.e., PGC1-α1 [34]) has been shown to be the master regulator of mitochondrial biogenesis in multiple tissues [32, 33]. Likewise, skeletal muscle over-expression of PGC1-α1 mimics many of the protective effects of exercise on multiple tissues [35, 36]. Interestingly, the newly identified splice variant of PGC1-α, PGC1-α4, has also been shown to induce skeletal muscle hypertrophy when transgenically overexpressed in mice consistent with a resistance-trained phenotype [34]. As such, efforts were made to identify the mechanism by which the expression and activity of PGC1-α in skeletal muscle affects other tissues. These studies were conducted under the backdrop of recent interest in skeletal muscle secreted signaling peptides referred to as myokines [37]. Could the increased expression of PGC1-α in skeletal muscle result in the secretion of some known or unknown set of myokines that affect the function of other tissues?

Bostrom et al. [14••] first examined the WAT depots of muscle specific PGC1-α transgenic mice to determine if there were molecular and/or metabolic differences in WAT compared to their wild-type littermates. They demonstrated that the inguinal WAT (subcutaneous WAT) consisted of a population of beige/brite adipocytes that had increased levels of UCP1 and Cidea [14••] in muscle specific PGC1-α transgenic mice compared to their wild-type littermates. Next, they demonstrated that wheel running and swimming substantially induced the expression of UCP1 in inguinal WAT (~25 fold, ~65-fold, respectively) [14••]. Taken together, these findings indicate that increased expression of PGC1-α in murine skeletal muscle induces browning of inguinal WAT, similar to what is observed through traditional exercise regimens. To address whether this browning of the subcutaneous WAT was due directly to muscle-fat cell signaling (e.g., myokine stimulated browning of WAT) they next cultured primary murine subcutaneous adipocytes with conditioned media from PGC1-α over expressing murine myocytes. Again, they found that the conditioned media treated murine subcutaneous adipocytes had increased expression brown-fat-specific genes (e.g., UCP1 and Cidea) [37]. These findings suggest that PGC1-α overexpressing murine myocytes secrete one or more myokines capable of inducing a thermogenic program in murine subcutaneous adipocytes.

Bostrom et al. [14••] used a combination of gene array technology and advanced bioinformatic algorithms to predict potential proteins that could be secreted by skeletal muscle and induce browning of WAT. Fibronectin type III domain-containing 5 (FNDC5) was one of five target genes of PGC1-α which could be secreted [14••]. Importantly, FNDC5 expression was increased in muscle from exercise trained mice as well as humans [14••]. Next, they demonstrated that the treatment of primary subcutaneous adipocytes during differentiation with recombinant-FNDC5 increased the expression of BAT genes (UCP1, Elovl3, Cox7a, and Otop1) [14••]. Moreover, recombinant-FNDC5 treated UCP1-positive cells also developed multilocular lipid droplets and increased mitochondrial content [14••]. Importantly, high-resolution respirometry experiments revealed that the recombinant-FNDC5 treated UCP1-positive cells had increased oxygen consumption, particularly with respect to uncoupled respiration [14••]. Taken together, the authors concluded that these findings indicate that FNDC5 induces UCP1-positive cells to develop the beige/brite phenotype in mice [14••]. However, it should be noted that the recombinant-FNDC5 protein used in these experiments had a truncated sequence [14••, 38••]. One potential mechanism by which FNDC5 induces browning of subcutaneous adipocytes is through increasing the expression of PPAR-α [14••]. Indeed, treatment of primary subcutaneous adipocytes with a PPAR-α antagonist attenuated the browning effect of the recombinant-FNDC5 [14••].

Before the protein product of FNDC5 was termed “irisin” [14••], it had already been discovered by two independent groups and assigned the gene aliases “PeP” [39] and “frcp2” [40]. PeP and frcp2 were both found to be expressed in skeletal muscle, heart, and brain of adult mice [39, 40]. It should be noted, however, that PeP and frcp2 expression in adipose tissue was not assessed [39, 40]. Bostrom et al. [14••] were the first to recognize and suggest that although the full-length FNDC5 is a trans-membrane protein, its extracellular N-terminal portion of FNDC5 could potentially be cleaved by a yet to be identified protease. Identification of the FNDC5 fragment was initially determined by antibody binding, which was confirmed by mass spectrometry [14••]. Their analysis also revealed that the secreted form of FNDC5 was highly homologous between mouse and humans [14••]. They named this newly identified signaling peptide (myokine) irisin after the messenger goddess of ancient Hellenic mythology, Iris [14••]. Another potential mechanism that has been suggested for observing N-terminal (extracellular) fragments of FNDC5 (e.g., irisin) in cell culture media and/or plasma *in vivo* is through shedding of the extracellular fraction [38••].

Using an antibody against FNDC5 Bostrom et al. [14••] were able to reduce the browning effect of media conditioned by skeletal muscle PGC1-α overexpressing murine myocytes on primary subcutaneous fat. Of note, the antibody used in these experiments was targeted at the c-terminus of FNDC5, which should not be present in irisin. They also indicated that irisin was present in both mouse and human plasma [14••]. Moreover, they demonstrate that plasma irisin concentrations were elevated in mice and older humans after short-term exercise training [14••]. Using adenoviral delivery of FNDC5 to the liver they were also able to increase plasma concentration of irisin which led to the browning of subcutaneous WAT while protecting against diet induced obesity and insulin-resistance [14••]. The increased circulating levels of irisin were also associated with increased expression of mitochondrial genes in the subcutaneous WAT and a concomitant increase in oxygen consumption. Taken together these data indicate that irisin, which is secreted from active (murine) skeletal muscle has the potential to protect against obesity and insulin resistance. However, it remains to be demonstrated that irisin is secreted from human skeletal muscle, and if not so to determine whether the potential action of FNDC5 and/or irisin is derived from non-skeletal muscle tissue.

## Does Irisin affect Human White Adipocytes

Several manuscripts have demonstrated that irisin enhances the “browning” of white adipocytes in mice [14••, 15], particularly in white adipocytes that highly express CD137 [15]. However, recent evidence has begun to question the physiological relevance of irisin in humans [41••, 42••]. Can the results obtained in mice be readily translated to humans? Specifically, is irisin synthesized and secreted from active human skeletal muscle? Is skeletal muscle the primary source of irisin and/or FNDC5? Can irisin induce browning of white adipocytes in humans?

As previously discussed, Bostrom et al. [14••] reported that 10 weeks of combined endurance plus resistance exercise training increases circulating levels of irisin in older adults approximately two-fold, which was proportional to the increase in skeletal muscle mRNA expression of FNDC5. However, Timmons et al. [42••] demonstrated that neither endurance training nor resistance training increases skeletal muscle FNDC5 mRNA expression in healthy adults. Nevertheless, they did report that FNDC5 expression was elevated in a subset of exercise trained older adults but not younger adults compared to their sedentary counterparts, which is an interesting finding [42••]. Raschke et al. [41••] also failed to demonstrate an increase in FNDC5 expression using an *in vitro* model of endurance training (electrical pulse stimulation) in human myotubes as well as in response to aerobic interval training and strength training in sedentary males. Results of a recently published randomized clinical trial of (*n* = 102) middle aged (30-60 years old) participants demonstrated that neither endurance training nor resistance training increase circulating irisin concentrations after 26 weeks of training compared to controls [43•]. An important observation from this study was that irisin is prone to storage-related degradation [43•]. Therefore, time related changes in circulating irisin concentrations in the absence of timed-matched controls should be interpreted with caution. In another study, it was demonstrated that an acute bout of endurance exercise, chronic endurance exercise, and chronic endurance combined with resistance exercise provide conflicting results with respect to skeletal muscle PGC1-α expression, FNDC5 expression, and circulating irisin [44•]. Twelve weeks of exercise training has also been reported to have little to no effect on genes expression in subcutaneous WAT for genes associated with browning of WAT (e.g., UCP1, PRDM16, TBX1, TMEM26, or CD137), despite significant increases in skeletal muscle FNDC5 [45••]. Taken together, the results of Timmons et al. [42•], Raschke et al. [41••], Hacksteden et al. [43•], Pekkela et al. [44•], and Norheim et al. [45••] raise significant concerns regarding the muscle-specific effects of exercise training on the stimulation of irisin in humans.

The human gene sequence of FNDC5 is also raising concerns regarding the translated product [46]. Although the FNDC5 gene sequence is highly conserved across species [14••], the human FNDC5 gene has a variation in its start codon that could essentially affect its translation [46]. Specifically, the translational initiation “ATG” codon is mutated to “ATA” resulting in substantial reduction in the translational efficiency of FNDC5 and leading to the translation of only 1 % of full-length FNDC5 as reported [41••]. Furthermore, there is a truncated isoform of FNDC5 that is translated by a downstream “ATG” start codon that is lacking the signaling peptide [41••]. Consistent with this later finding, on September 5th 2012, the UniProt database was annotated to include a second, truncated, protein sequence for FNDC5 [41••]. Raschke et al. [41••] further demonstrated that neither recombinant-irisin nor recombinant-FNDC5 induces “browning” of human pre-adipocytes. In contrast, they demonstrated that BMP7 (i.e., the positive control) resulted in an activation of the genes regulating the “browning” of human pre-adipocytes characterized by elevations in PPARγ, UCP1, PGC-1β, as well as an elevation in mitochondrial protein content [41••]. Taken together, these findings indicate that the ability of endogenous irisin to stimulate “browning” of white adipocytes in humans remains to be proven.

Surprisingly, little information is known about the expression and potential secretion of FNDC5/irisin from adipose tissue itself. A few recent papers, however, highlight the presence of FNDC5 in rat and human WAT [47, 48••, 49]. Using gene-array technology our research team recently demonstrated that FNDC5 is highly expressed in visceral adipose tissue, epigastric adipose tissue, and to a lesser extent subcutaneous adipose tissue of severely obese patients undergoing bariatric surgery [48••]. Likewise, PGC1-α was also abundantly expressed in these adipose tissue depots [48••]. Taken together, it appears that human WAT has some of the key components necessary for FNDC5-induced browning WAT in an autocrine fashion in humans. In another study, it was demonstrated by real-time PCR that FNDC5 gene expression was reduced in obese as well as patients with type 2 diabetes [49]. Moreover, FNDC5 gene expression in visceral and subcutaneous WAT was positively associated with brown adipose tissue markers (PRDM16 and UCP1) in humans [49]. Future investigations are warranted to examine the potential role of FNDC5 to induce browning of WAT in humans via autocrine mechanisms. However, it has been reported that the expression of FNDC5 in WAT is less than five percent of that observed in skeletal muscle in humans [49, 50]. Roca-Rivada et al. [47] recently reported that WAT explants secrete FNDC5/irisin, which is increased after 1 week of exercise in rats [47]. Interestingly, however, the secretion of FNDC5/irisin from the WAT explants was reduced after 3 weeks of exercise training [47]. The secretion of FNDC5/irisin was based on quantification of the 25 kDa band (predicted MW of irisin ~12 kDa), which was detected both by the Abcam and the Phoenix antibodies [47]. Moreover, the Abcam FNDC5 antibody recognizes the C-terminus of FNDC5 that is not supposed to be part of irisin [38••]. It should also be noted, the Abcam and the Phoenix antibodies do not share any sequence overlap [38••]. In summary, it appears that human WAT has some of the key components necessary for FNDC5 to act in an autocrine fashion to brown WAT in humans. However, future studies are needed using validated antibodies to determine whether human WAT secretes full-length FNDC5 and/or irisin. Likewise, experimental evidence demonstrating the ability of FNDC5/irisin to brown human WAT resulting in increased UCP1 expression and thermogenesis regardless of its source is currently lacking.

## Conclusion

**Fig. 1:**
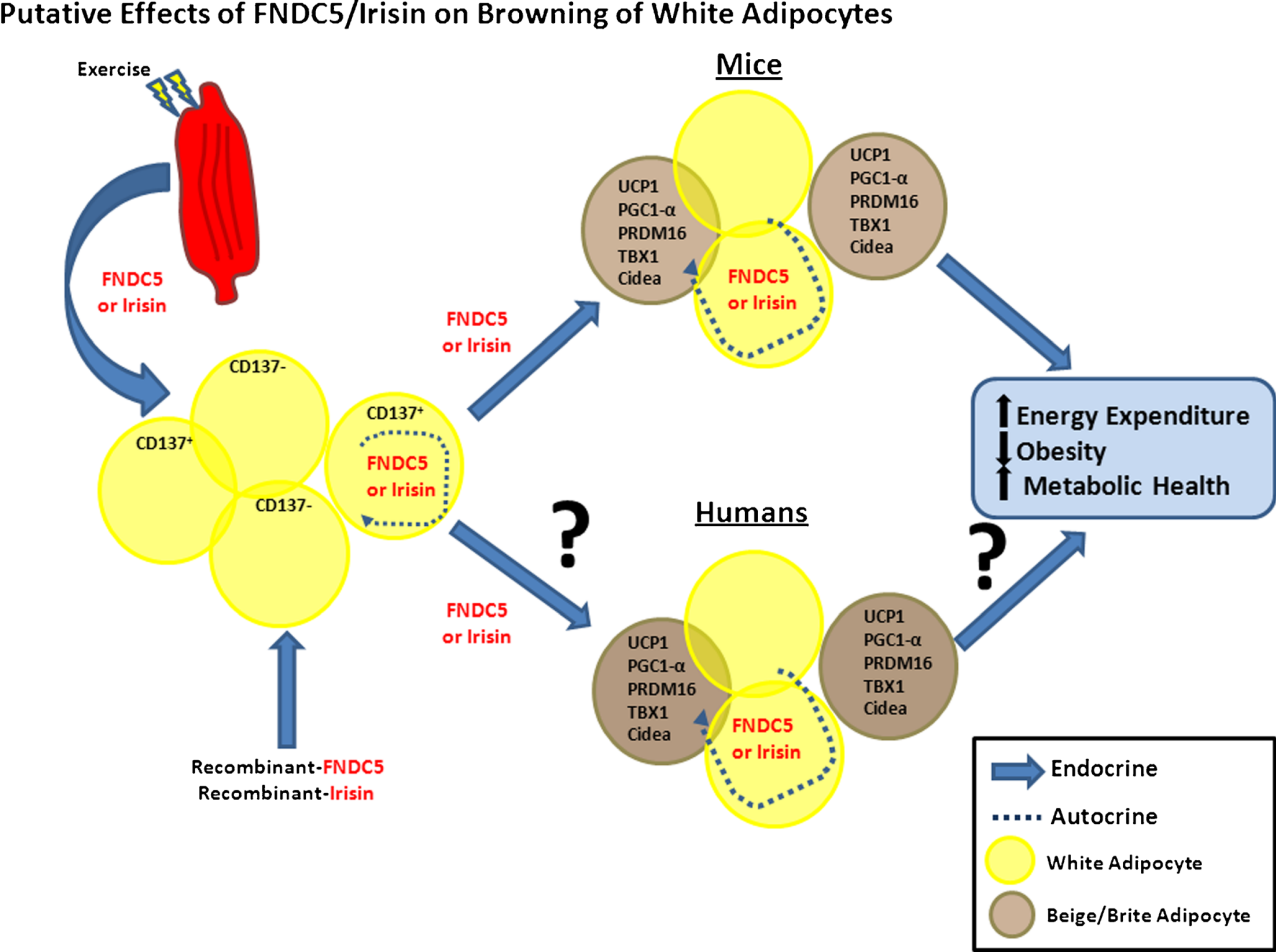
Putative effects of FNDC5/irisin on browning of white adipocytes and improvements in energy expenditure, obesity, and metabolic health in mice and humans

The recent discovery of irisin has garnered much attention as a potential therapeutic agent for the treatment of obesity and its comorbid conditions. Figure 1 presents the putative effects of irisin on the browning of murine and human white adipocytes. Preliminary studies have indicated that recombinant FNDC5 and/or irisin can induce browning of murine WAT. However, there remains a dearth of evidence to indicate that recombinant FNDC5 and/or irisin can induce browning of human WAT. Moreover, recent studies in humans also indicate that neither acute nor chronic exercise consistently results in increased expression of endogenous FNDC5 and/or increased circulating concentrations of FNDC5/irisin in humans. These later findings may be due in part to the lack of available antibodies that are validated to detect FNDC5/irisin. In addition, care should be taken when extrapolating data derived from mouse studies to human physiology given the significant differences in the abundance of brown fat between humans and mice. Finally, the expression of FNDC5 in human WAT opens the door to the possibility that FNDC5 acts in an autocrine fashion to brown WAT in humans.
